# Fast algorithms for approximate circular string matching

**DOI:** 10.1186/1748-7188-9-9

**Published:** 2014-03-22

**Authors:** Carl Barton, Costas S Iliopoulos, Solon P Pissis

**Affiliations:** 1 King’s College London, London, UK; 2 University of Western Australia, Crawley, Australia; 3 Curtin University, Bentley, Australia

**Keywords:** Approximate circular string matching, Circular pattern matching, Algorithms on strings

## Abstract

**Background:**

Circular string matching is a problem which naturally arises in many biological contexts. It consists in finding all occurrences of the rotations of a pattern of length *m* in a text of length *n*. There exist optimal average-case algorithms for exact circular string matching. Approximate circular string matching is a rather undeveloped area.

**Results:**

In this article, we present a suboptimal average-case algorithm for exact circular string matching requiring time

O(n)

. Based on our solution for the exact case, we present two fast average-case algorithms for approximate circular string matching with *k*-mismatches, under the Hamming distance model, requiring time

O(n)

for moderate values of *k*, that is

k=O(m/logm)

. We show how the same results can be easily obtained under the edit distance model. The presented algorithms are also implemented as library functions. Experimental results demonstrate that the functions provided in this library accelerate the computations by more than three orders of magnitude compared to a naïve approach.

**Conclusions:**

We present two fast average-case algorithms for approximate circular string matching with *k*-mismatches; and show that they also perform very well in practice. The importance of our contribution is underlined by the fact that the provided functions may be seamlessly integrated into any biological pipeline. The source code of the library is freely available at http://www.inf.kcl.ac.uk/research/projects/asmf/.

## Background

Circular sequences appear in a number of biological contexts. This type of structure occurs in the DNA of viruses [1,2], bacteria [3], eukaryotic cells [4], and archaea [5]. In [6], it was noted that, due to this, algorithms on circular strings may be important in the analysis of organisms with such structure. Circular strings have previously been studied in the context of sequence alignment. In [7], basic algorithms for pairwise and multiple circular sequence alignment were presented. These results were later improved in [8], where an additional preprocessing stage was added to speed up the execution time of the algorithm. In [9], the authors also presented efficient algorithms for finding the optimal alignment and consensus sequence of circular sequences under the Hamming distance metric.

In order to provide an overview of our results and algorithms, we begin with a few definitions, generally following [10]. We think of a *stringx* of *lengthn* as an array *x*[ 0..*n*−1], where every *x*[ *i*], 0≤*i*<*n*, is a *letter* drawn from some fixed *alphabetΣ* of size *σ*=|*Σ*|. The *empty string* of length 0 is denoted by *ε*. A string *x* is a *factor* of a string *y* if there exist two strings *u* and *v*, such that *y*=*uxv*. Let the strings *x*,*y*,*u*, and *v* be such that *y*=*uxv*. If *u*=*ε*, then *x* is a *prefix* of *y*. If *v*=*ε*, then *x* is a *suffix* of *y*.

Let *x* be a non-empty string of length *n* and *y* be a string. We say that there exists an *occurrence* of *x* in *y*, or, more simply, that *xoccurs iny*, when *x* is a factor of *y*. Every occurrence of *x* can be characterised by a position in *y*. Thus we say that *x* occurs at the *starting positioni* in *y* when *y*[ *i*..*i*+*n*−1]=*x*. The *Hamming distance* between strings *x* and *y*, both of length *n*, is the number of positions *i*, 0≤*i*<*n*, such that *x*[ *i*]≠*y*[ *i*]. Given a nonnegative integer *k*, we write *x*≡_*k*_*y* if the Hamming distance between *x* and y is at most *k*.

A circular string of length *n* can be viewed as a traditional linear string which has the left- and right-most symbols wrapped around and stuck together in some way. Under this notion, the same circular string can be seen as *n* different linear strings, which would all be considered equivalent. Given a string *x* of length *n*, we denote by *x*^*i*^=*x*[ *i*..*n*−1]*x*[0..*i*−1], 0<*i*<*n*, the *i*-th *rotation* of *x* and *x*^0^=*x*. Consider, for instance, the string *x*=*x*^0^=abababbc; this string has the following rotations: *x*^1^=bababbca, *x*^2^=ababbcab, *x*^3^=babbcaba, *x*^4^=abbcabab, *x*^5^=bbcababa, *x*^6^=bcababab, *x*^7^=cabababb.

Here we consider the problem of finding occurrences of a pattern string *x* of length *m* with circular structure in a text string *t* of length *n* with linear structure. For instance, the DNA sequence of many viruses has circular structure, so if a biologist wishes to find occurrences of a particular virus in a carriers DNA sequence—which may not be circular—they must consider how to locate all positions in *t* that at least one rotation of *x* occurs. This is the problem of *circular string matching*.

The problem of exact circular string matching has been considered in [11], where an O(n)-time algorithm was presented. A naïve solution with quadratic complexity consists in applying a classical algorithm for searching a finite set of strings after having built the *trie* of rotations of *x*. The approach presented in [11] consists in preprocessing *x* by constructing a *suffix automaton* of the string *xx*, by noting that every rotation of *x* is a factor of *xx*. Then, by feeding *t* into the automaton, the lengths of the longest factors of *xx* occurring in *t* can be found by the links followed in the automaton in time O(n). In [12], the authors presented an optimal average-case algorithm for exact circular string matching, by also showing that the average-case lower bound for single string matching of $O(n\underset{\sigma }{\log}m/m)$ also holds for circular string matching. Very recently, in [13], the authors presented two fast average-case algorithms based on word-level parallelism. The first algorithm requires average-case time $O(n\underset{\sigma }{\log}m/w)$, where *w* is the number of bits in the computer word. The second one is based on a mixture of word-level parallelism and *q*-grams. The authors showed that with the addition of *q*-grams, and by setting $q=O(\underset{\sigma }{\log}m)$, an optimal average-case time of $O(n\underset{\sigma }{\log}m/m)$ is achieved. Indexing circular patterns [14] and variations of approximate circular string matching under the edit distance model [15]—both based on the construction of a *suffix tree*—have also been considered.

In this article, we consider the following problems.

### **Problem 1** (Exact Circular String Matching).

Given a pattern *x* of length *m* and a text *t* of length *n*>*m*, find all factors *u* of *t* such that *u*=*x*^*i*^, 0≤*i*<*m*.

### **Problem 2** (Approximate Circular String Matching with *k*-Mismatches).

Given a pattern *x* of length *m*, a text *t* of length *n*>*m*, and an integer threshold *k*<*m*, find all factors *u* of *t* such that *u*≡_*k*_*x*^*i*^, 0≤*i*<*m*.

The aforementioned algorithms for the exact case exhibit the following disadvantages: first, they cannot be applied in a biological context since both single nucleotide polymorphisms as well as errors introduced by wet-lab sequencing platforms might have occurred in the sequences; second, it is not clear whether they could easily be adapted to deal with the approximate case. Similar to the exact case [12], it can be shown that the average-case lower bound for single approximate string matching of $O(n(k+\underset{\sigma }{\log}m)/m)$[16] also holds for approximate circular string matching with *k*-mismatches under the Hamming distance model. To the best of our knowledge, no optimal average-case algorithm exists for this problem. Therefore, to achieve optimality, one could use the optimal average-case algorithm for multiple approximate string matching, presented in [17], for matching the *r*=*m* rotations of *x* requiring, on average, time $O(n(k+\underset{\sigma }{\log}\text{rm})/m)$, only if $k/m<1/2-O(1/\sqrt{\sigma })$, $r=O(\min(n^{1/3}/m^{2},\sigma^{o(m)}))$, and we have $O(m^{4}r^{2}\sigma^{O(1)})$ space available; which is impractical for large *m*: e.g. the genome of the smallest known viruses replicating autonomously in eukaryotic cells is around 1.8 KB long. The authors propose solutions to reduce the required space, however using various space–time trade-off techniques.

**Our Contribution.** We present a new suboptimal average-case algorithm for exact circular string matching requiring time O(n). Although suboptimal, this algorithm can be easily extended to tackle the approximate case efficiently. Based on our solution for the exact case, we present two new fast average-case algorithms for approximate circular string matching with *k*-mismatches, under the Hamming distance model, requiring time O(n) for moderate values of *k*, that is $k=O(m/\underset{\sigma }{\log}m)$. The first algorithm requires space O(n) and the second one O(m). We show how the same results can be easily obtained under the edit distance model. The presented algorithms are also implemented as library functions. Experimental results demonstrate that the functions provided in this library accelerate the computations by more than three orders of magnitude compared to a naïve approach. The source code of the library is freely available at http://www.inf.kcl.ac.uk/research/projects/asmf/.

## Properties of the partitioning technique

In this section, we give a brief outline of the *partitioning* technique in general; and then show some properties of the version of the technique we use for our algorithms. The partitioning technique, introduced in [18], and in some sense earlier in [19], is an algorithm based on *filtering out* candidate positions that could never give a solution to speed up string-matching algorithms. An important point to note about this technique is that it reduces the search space but does not, by design, verify potential occurrences. To create a string-matching algorithm filtering must be combined with some verification technique. The idea behind the partitioning technique was initially proposed for approximate string matching, but here we show that this can also be used for exact circular string matching.

The idea behind the partitioning technique is to partition the given pattern in such a way that at least one of the fragments must occur exactly in any valid approximate occurrence of the pattern. It is then possible to search for these fragments exactly to give a set of *candidate* occurrences of the pattern. It is then left to the verification portion of the algorithm to check if these are valid approximate occurrences of the pattern. It has been experimentally shown that this approach yields very good practical performance on large-scale datasets [20], even if it is not theoretically optimal.

For exact circular string matching, for an efficient solution, we cannot simply apply well-known exact string-matching algorithms, as we must also take into account the rotations of the pattern. We can, however, make use of the partitioning technique and, by choosing an appropriate number of fragments, ensure that at least one fragment must occur in any valid exact occurrence of a rotation. Lemma 1 together with the following fact provide this number.

### 

**Fact 1**. *Any rotation ofx*=*x*[ 0..*m*−1] *is a factor ofx*^′^=*x*[ 0..*m*−1]*x*[ 0..*m*−2]; *and any factor of lengthm* of *x*^′^*is a rotation ofx*.

### 

**Lemma 1**. *If we partitionx*^′^=*x*[ 0..*m*−1]*x*[ 0.. *m*−2] *in 4 fragments of length* ⌊(2*m*−1)/4⌋ and ⌈(2*m*−1)/4⌉, *at least one of the 4 fragments is a factor of any factor of lengthm* of *x*^′^.

**Figure 1:**
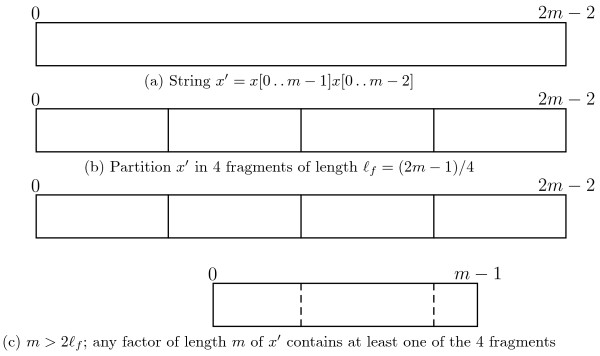
**Lemma **1**.** Illustration of Lemma 1.

*Proof.* Let *ℓ*_*f*_ denote the length of the fragment. If we partition *x*^′^ in at least 4 fragments of length ⌊(2*m*−1)/4⌋ and ⌈(2*m*−1)/4⌉, we have that

$$\ell_{f}\leq (2m-1)/4,$$

which gives 2*m*>4*ℓ*_*f*_ and *m*>2*ℓ*_*f*_. Therefore any factor of length *m* of *x*^′^, and, by Fact 1, any rotation of *x*, must contain at least one of the fragments. For a graphical illustration of this proof inspect Figure 1. ■

### 

**Lemma 2**. *Letxandy*=*y*_0_*y*_1_…*y*_*k*_*be two strings, both of lengthn*, *such thaty*_0_,*y*_1_,…,*y*_*k*_*arek*+1≤*nnon-empty strings andx*≡_*k*_*y*. *Then there exists at least one stringy*_*i*_, 0≤*i*≤*k*, *starting at positionj* of *y*, 0≤*j*<*n*, *occurring at the starting positionj* of *x*.

*Proof.* Immediate from the pigeonhole principle—if *n* items are put into *m*<*n* pigeonholes, then at least one pigeonhole must contain more than one item. ■

Based on Lemma 2, we take a similar approach to the one described by Lemma 1, to obtain the sufficient number of fragments in the case of approximate circular string matching with *k*-mismatches.

### 

**Lemma 3**. *If we partitionx*^′^=*x*[ 0.. *m*−1]*x*[ 0.. *m*−2] in 2*k*+4 *fragments of length* ⌊(2*m*−1)/(2*k*+4)⌋ *and* ⌈(2*m*−1)/(2*k*+4)⌉, *at leastk*+1 *of the* 2*k*+4 *fragments are factors of any factor of lengthm* of *x*^′^.

**Figure 2:**
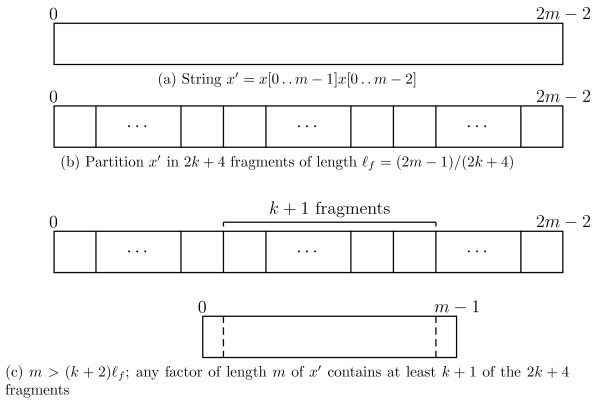
**Lemma **3**.** Illustration of Lemma 3.

*Proof.* Let *ℓ*_*f*_ denote the length of the fragment. If we partition *x*^′^ in 2*k*+4 fragments of length ⌊(2*m*−1)/(2*k*+4)⌋ and ⌈(2*m*−1)/(2*k*+4)⌉, we have that

$$\ell_{f}\leq (2m-1)/(2k+4),$$

which gives 2*m*−1≥2(*k*+2)*ℓ*_*f*_ and *m*>(*k*+2)*ℓ*_*f*_. Therefore any factor of length *m* of *x*^′^, and, by Fact 1, any rotation of *x*, must contain at least *k*+1 of the fragments. For a graphical illustration of this proof inspect Figure 2. ■

## Exact circular string matching via filtering

In this section, we present ECSMF, a new suboptimal average-case algorithm for exact circular string matching via filtering. It is based on the partitioning technique and a series of practical and well-established data structures such as the suffix array (for more details see [21]).

### Longest common extension

First, we describe how to compute the longest common extension, denoted by lce, of two suffixes of a string in constant time (for more details see [22]). lce queries are an important part of the algorithms presented later on.

Let SA denote the array of positions of the sorted suffixes of string *x* of length *n*, i.e. for all 1≤*r*<*n*, we have *x*[ SA[ *r*−1]..*n*−1]<*x*[ SA[ *r*]..*n*−1]. The inverse iSA of the array SA is defined by iSA[ SA[ *r*]]=*r*, for all 0≤*r*<*n*. Let lcp(*r*,*s*) denote the length of the longest common prefix of the strings *x*[ SA[ *r*].. *n*−1] and *x*[ SA[ *s*].. *n*−1], for all 0≤*r*,*s*<*n*, and 0 otherwise. Let LCP denote the array defined by LCP[ *r*]=lcp(*r*−1,*r*), for all 1<*r*<*n*, and LCP[ 0]=0. We perform the following linear-time and linear-space preprocessing: 

• Compute arrays SA and iSA of *x*[21].

• Compute array LCP of *x*[23].

• Preprocess array LCP for range minimum queries, we denote this by RMQ_LCP_[24].

With the preprocessing complete, the lce of two suffixes of *x* starting at positions *p* and *q* can be computed in constant time in the following way [22]:

$$\text{LCE}(x,p,q)=\text{LCP}[\;{\text{RMQ}}_{\text{LCP}}(\text{iSA}[\;p]+1,\text{iSA}[\;q])].$$

#### 

**Example 1**. *Let the stringx=abbababba. The following table illustrates the arrays SA, iSA, and LCP forx*. 

*We have LCE*(*x*,1,2)=LCP[ RMQ_LCP_(iSA[ 2]+1,iSA[ 1])]=LCP[ RMQ_LCP_(6,8)]=1, *implying that the*lce*of*bbababba*and*bababba*is 1.*

### Algorithm ECSMF

Given a pattern *x* of length *m* and a text *t* of length *n*>*m*, an outline of algorithm ECSMF for solving Problem 1 is as follows.

1. Construct the string *x*^′^=*x*[ 0.. *m*−1]*x*[ 0.. *m*−2] of length 2*m*−1. By Fact 1, any rotation of *x* is a factor of *x*^′^.

2. The pattern *x*^′^ is partitioned in 4 fragments of length ⌊(2*m*−1)/4⌋ and ⌈(2*m*−1)/4⌉. By Lemma 1, at least one of the 4 fragments is a factor of any rotation of *x*.

3. Match the 4 fragments against the text *t* using an Aho Corasick automaton [25]. Let be a list of size *Occ* of tuples, where $<p_{x^{\prime }},\ell ,p_{t}>\in ℒ$ is a 3-tuple such that $0\leq p_{x^{\prime }}<2m-1$ is the position where the fragment occurs in *x*^′^, *ℓ* is the length of the corresponding fragment, and 0≤*p*_*t*_<*n* is the position where the fragment occurs in *t*.

4. Compute SA, iSA, LCP, and RMQ_LCP_ of *T*=*x*^′^*t*. Compute SA, iSA, LCP, and RMQ_LCP_ of *T*_*r*_=rev(*tx*^′^), that is the reverse string of *tx*^′^.

5. For each tuple $<p_{x^{\prime }},\ell ,p_{t}>\in ℒ$, we try to extend to the right via computing

$$E_{r}\leftarrow \text{LCE}(T,p_{x^{\prime }}+\ell ,2m-1+p_{t}+\ell );$$

in other words, we compute the length $E_{r}$ of the longest common prefix of $x^{\prime }[\;p_{x^{\prime }}+\ell \;.\;.\;2m-1]$ and *t*[ *p*_*t*_+*ℓ*.. *n*−1], both being suffixes of *T*. Similarly, we try to extend to the left via computing $E_{l}$ using lce queries on the suffixes of *T*_*r*_.

6. For each $E_{l},E_{r}$ computed for tuple $<p_{x^{\prime }},\ell ,p_{t}>\in ℒ$, we report all the valid starting positions in *t* by first checking if the total length $E_{l}+\ell +E_{r}\geq m$; that is the length of the full extension of the fragment is greater than or equal to *m*, matching at least one rotation of *x*. If that is the case, then we report positions

$$\max\{p_{t}-E_{\ell },p_{t}+\ell -m\},\ldots ,\min\{p_{t}+\ell -m+E_{r},p_{t}\}.$$

#### **Example 2**.

Let the pattern *x*=GGGTCTA of length *m*=7, and the text *t*=GATACGATACCTAGGGTGATAGAATAG. Then *x*^′^=GGGTCTAGGGTCT (Step 1). *x*^′^ is partitioned in GGGT, CTA, GGG, and TCT (Step 2). Consider <4,3,10>∈ℒ, that is, fragment *x*^′^[ 4..6]=CTA, of length *ℓ*=3, occurs at starting position *p*_*t*_=10 in *t* (Step 3). Then *T*=GGGTCTAGGGTCTGATACGATACCTAGGGTGATAGAATAG and *T*_*r*_=TCTGGGATCTGGGGATAAGATAGTGGGATCCATAGCATAG (Step 4). Extending to the left gives $E_{l}=0$, since *T*_*r*_[ 9]≠*T*_*r*_[ 30]; and extending to the right gives $E_{r}=4$, since *T*[ 7..10]=*T*[ 26..29] and *T*[ 11]≠*T*[ 30] (Step 5). We check that $E_{l}+\ell +E_{r}=7=m$, and therefore we report position 10 (Step 6):

$$p_{t}-E_{\ell }=10-0=10,\ldots ,p_{t}+\ell -m+E_{r}=10+3-7+4=10;$$

that is, *x*^4^=CTAGGGT occurs at starting position 10 in *t*.

#### 

**Theorem 1**. *Given a patternxof lengthmdrawn from alphabetΣ*, *σ*=|*Σ*|, *and a texttof lengthn*>*mdrawn fromΣ*, *algorithm*ECSMF*requires average-case time*O(n) to solve Problem 1.

*Proof.* Constructing and partitioning the string *x*^′^ from *x* can trivially be done in time O(m) (Step 1-2). Building the Aho-Corasick automaton of the 4 fragments requires time O(m); and the search time is O(n+Occ) (Step 3) [25]. The preprocessing step for the lce queries on the suffixes of *T* and *T*_*r*_ can be done in time O(n) (Step 4). Computing $E_{l}$ and $E_{r}$ for each occurrence of a fragment requires time O(Occ) (Step 5). For each extended occurrence of a fragment, we report O(m) valid starting positions, thus O(mOcc) in total (Step 6). Since the expected number *Occ* of occurrences of the 4 fragments in *t* is $4n/\sigma^{(2m-1)/4}=O(\frac{n}{\sigma^{\frac{2m-1}{4}}})$, algorithm ECSMF requires average-case time $O((1+\frac{m}{\sigma^{\frac{2m-1}{4}}})n)$. It achieves average-case time O(n)*iff*

$$f=\frac{4m}{\sigma^{\frac{2m-1}{4}}}n\leq \text{cn}$$

for some fixed constant *c*. For *σ*=2, the maximum value of *f* is attained at

m=2/ln2≈2.8853

and so for *σ*>1 we get

$$\frac{4m}{\sigma^{\frac{2m-1}{4}}}n\leq 5.05\mathrm{n.}$$

■

## Approximate circular string matching with *k*-mismatches via filtering

In this section, based on the ideas presented in algorithm ECSMF, we present algorithms ACSMF and ACSMF-Simple, two new fast average-case algorithms for approximate circular string matching with *k*-mismatches via filtering.

### Algorithm ACSMF

The first four steps of algorithm ACSMF are essentially the same as in algorithm ECSMF. A small difference exists in Step 2, where the sufficient number of fragments in the case of approximate circular string matching with *k*-mismatches is used. The main difference is in Step 5, where algorithm ACSMF tries to extend *k*+1 times to the right and *k*+1 times to the left. Given a pattern *x* of length *m*, a text *t* of length *n*>*m*, and an integer threshold *k*<*m*, an outline of algorithm ACSMF for solving Problem 2 is as follows.

1. Construct the string *x*^′^=*x*[ 0.. *m*−1]*x*[0.. *m*−2] of length 2*m*−1. By Fact 1, any rotation of *x* is a factor of *x*^′^.

2. The pattern *x*^′^ is partitioned in 2*k*+4 fragments of length ⌊(2*m*−1)/(2*k*+4)⌋ and ⌈(2*m*−1)/(2*k*+4)⌉. By Lemma 3, at least *k*+1 of the 2*k*+4 fragments are factors of any rotation of *x*.

3. Match the 2*k*+4 fragments against the text *t* using an Aho Corasick automaton [25]. Let be a list of size *Occ* of tuples, where $<p_{x^{\prime }},\ell ,p_{t}>\in ℒ$ is a 3-tuple such that $0\leq p_{x^{\prime }}<2m-1$ is the position where the fragment occurs in *x*^′^, *ℓ* is the length of the corresponding fragment, and 0≤*p*_*t*_<*n* is the position where the fragment occurs in *t*.

4. Compute SA, iSA, LCP, and RMQ_LCP_ of *T*=*x*^′^*t*. Compute SA, iSA, LCP, and RMQ_LCP_ of *T*_*r*_=rev(*tx*^′^), that is the reverse string of *tx*^′^.

5. For each tuple $<p_{x^{\prime }},\ell ,p_{t}>\in ℒ$, we try to extend *k*+1 times to the right via computing

$$E_{r}^{0}\leftarrow \text{LCE}(T,p_{x^{\prime }}+\ell ,2m-1+p_{t}+\ell )+1$$

$$\begin{array}{c} E_{r}^{1}\leftarrow \text{LCE}(T,p_{x^{\prime }}+\ell +E_{r}^{0},2m-1+p_{t}+\ell +E_{r}^{0})+1\\ \ldots \end{array}$$

$$E_{r}^{k-1}\leftarrow \text{LCE}(T,p_{x^{\prime }}+\ell +E_{r}^{k-2},2m-1+p_{t}+\ell +E_{r}^{k-2})+1$$

$$E_{r}^{k}\leftarrow \text{LCE}(T,p_{x^{\prime }}+\ell +E_{r}^{k-1},2m-1+p_{t}+\ell +E_{r}^{k-1});$$

in other words, we compute the length $E_{r}^{k}$ of the longest common prefix of $x^{\prime }[\;p_{x^{\prime }}+\ell \;.\;.\;2m-1]$ and *t*[ *p*_*t*_+*ℓ*.. *n*−1], both being suffixes of *T*, with *k* mismatches. Similarly, we try to extend to the left *k*+1 times via computing $E_{l}^{k}$ using lce queries on the suffixes of *T*_*r*_.

6. For each tuple $<p_{x^{\prime }},\ell ,p_{t}>\in ℒ$ we try to extend, we also maintain an array M of size 2*m*−1, initialised with zeros, where we mark the position of the *i*-th left and right mismatch, 1≤*i*≤*k*, by setting

$$\text{M}[\;p_{x^{\prime }}-E_{l}^{i-1}-1]\leftarrow 1\;\;\text{and}\;\;\text{M}[\;p_{x^{\prime }}+\ell +E_{r}^{i-1}]\leftarrow 1.$$

7. For each $E_{l}^{k},E_{r}^{k},\text{M}$ computed for tuple $<p_{x^{\prime }},\ell ,p_{t}>\in ℒ$, we report all the valid starting positions in *t* by first checking if the total length $E_{l}^{k}+\ell +E_{r}^{k}\geq m$; that is the length of the full extension of the fragment is greater than or equal to *m*. If that is the case, then we count the total number of mismatches of the occurrences at starting positions

$$\max\{p_{t}-\overset{k}{\underset{\ell }{E}},p_{t}+\ell -m\},\ldots ,\min\{p_{t}+\ell -m+\overset{k}{\underset{r}{E}},p_{t}\},$$

by first summing up the mismatches for the leftmost starting position

$$\mu_{j}\leftarrow \text{M}[p_{x^{\prime }}-E_{l}^{k}]+\ldots +\text{M}[\;p_{x^{\prime }}-E_{l}^{k}+m-1],$$

$$\text{where}j=\max\{p_{t}-\overset{k}{\underset{\ell }{E}},p_{t}+\ell -m\}.$$

For each subsequent position *j*+1, we subtract the value of the leftmost element of M computed for *μ*_*j*_ and add the value of the next element to compute *μ*_*j*+1_. In case *μ*_*j*_≤*k*, we report position *j*.

#### 

**Example 3**. *Let the patternx=GGGTCTA of lengthm*=7, the text *t= GATACGATACCTAGGGTGATAGAATAG, andk=1. Thenx*^′^*= GGGTCTAGGGTCT (Step 1).x*^′^*is partitioned in GGG, TC, TA, GG, GT, and CT (Step 2). Consider*<9,2,15>∈ℒ, *that is, fragmentx*^′^*[ 9.. 10]=GT, of lengthℓ= 2, occurs at starting positionp*_*t*_=15 in *t* (*Step 3). ThenT=GGGTCTAGGGTCTGATACGATACCTAGGGTGATAGAATAG andT*_*r*_*=TCTGGGATCTGGGGATAAGATAGTGGGATCCATAGCATAG (Step 4). Extending to the left gives*$E_{l}^{k}=6$, since *T*_*r*_[ 4.. 9]≡_*k*_*T*_*r*_[ 25.. 30] and *T*_*r*_[ 10]≠*T*_*r*_[ 31];* and extending to the right gives*$E_{r}^{k}=1$, *sinceT*[ 11]≡_*k*_*T*[ 30] *andT*[ 12]≠*T[ 31] (Step 5). We also set M[ 3]=1 and M[ 11]=1 (Step 6). We check that*$E_{l}+\ell +E_{r}=9>m$, *and therefore we report positions 10, since*$\overset{10}{\underset{i=4}{\sum }}\text{M}[\;i]=0<k$, *and 11, since*$\overset{11}{\underset{i=5}{\sum }}\text{M}[\;i]=1=k$*(Step 7):*

$$\begin{array}{lll} p_{t}\; & +\;\ell \;-\;m\;=\;15\;+\;2\;-\;7\;=\;10\;,\ldots ,\; & \;\\ p_{t}\; & +\;\ell \;-\;m\;+\;E_{r}\;=\;15\;+\;2\;-\;7\;+\;1\;=\;11;\; & \; \end{array}$$

*that is,x*^4^*=CTAGGGT andx*^5^*=TAGGGTC occur at starting position 10 intwith no mismatch and at starting position 11 intwith 1 mismatch, respectively.*

#### 

**Theorem 2**. *Given a patternxof lengthmdrawn from alphabetΣ*, *σ*=|*Σ*|, *a texttof lengthn*>*mdrawn fromΣ*, *and an integer thresholdk*<*m*, *algorithm*ACSMF*requires average-case time*$O((1+\frac{\text{km}}{\sigma^{\frac{2m-1}{2k+4}}})n)$*and space*O(n)*to solve Problem 2.*

*Proof.* Constructing and partitioning the string *x*^′^ from *x* can trivially be done in time O(m) (Step 1-2). Building the Aho-Corasick automaton of the 2*k*+4 fragments requires time O(m); and the search time is O(n+Occ) (Step 3) [25]. The preprocessing step for the lce queries on the suffixes of *T* and *T*_*r*_ can be done in time and space O(n) (Step 4)—see Section 3. Computing $E_{l}^{k}$ and $E_{r}^{k}$ for each occurrence of a fragment requires time O(kOcc) (Step 5)—see Section 3. Maintaining array M is of no extra cost (Step 6). For each extended occurrence of a fragment, we report O(m) valid starting positions, thus O(mOcc) in total (Step 7). Since the expected number *Occ* of occurrences of the 2*k*+4 fragments is $\left(2k+4)n/\sigma^{\left(2m-1)/(2k+4\right)}=O(\frac{\text{kn}}{\sigma^{\frac{2m-1}{2k+4}}}\right)$, algorithm ACSMF requires average-case time $O((1+\frac{\text{km}}{\sigma^{\frac{2m-1}{2k+4}}})n)$ and space O(n). ■

#### 

**Corollary 1**. *Given a patternxof lengthmdrawn from alphabetΣ*, *σ*=|*Σ*|, *a texttof lengthn*>*mdrawn fromΣ*, *and an integer threshold*$k=O(m/\underset{\sigma }{\log}m)$, *algorithm*ACSMF*requires average-case time*O(n).

*Proof.* Algorithm ACSMF achieves average-case time O(n)*iff*

$$m(2k+4)n/\sigma^{\left(2m-1)/(2k+4\right)}\leq \text{cn}$$

for some fixed constant *c*. Let *r*=(2*m*−1)/(2*k*+4). We have

$$m(2k+4)n/\sigma^{r}\leq \mathrm{cn.}$$

Since *k*<*m*, we can (pessimistically) replace *k* by *m*−1. Then we have

$$2m(m+1)n/\sigma^{r}\leq \mathrm{cn.}$$

Solving for *r*, and using *k*≤(2*m*−1)/2*r*−2, gives the maximum value of *k*, that is

$$k=O(m/\underset{\sigma }{\log}m).$$

■

### Algorithm ACSMF-simple

Algorithm ACSMF-simple is very similar to Algorithm ACSMF. The only differences are: 

• Algorithm ACSMF-simple does not perform Step 4 of Algorithm ACSMF;

• For each tuple $<p_{x^{\prime }},\ell ,p_{t}>\in ℒ$, Step 5 of Algorithm ACSMF is performed without the use of the pre-computed indexes. In other words, we compute $E_{r}^{k}$ and $E_{\ell }^{k}$ by simply performing letter comparisons and counting the number of mismatches occurred. The extension stops right before the *k*+1th mismatch.

#### 

**Fact 2**. *The expected number of letter comparisons required for each extension in algorithm*ACSMF-simple*is less than 3.*

*Proof.* Recall that on an alphabet of size *σ*, the probability that two random strings of length *ℓ* are equal is (1/*σ*)^*ℓ*^. Thus, given two long strings, and setting *r*=1/*σ*, there is probability *r* that the initial letters are equal, *r*^2^ that the prefixes of length two are equal, and so on. Thus the expected number of positions to be matched before inequality occurs is

$$S=r+2r^{2}+\cdots +(n-1)r^{n-1},$$

for some *n*≥2. Hall & Knight [26, p. 44] tell us that

$$S=r(1-r^{n-1})/{\left(1-r\right)}^{2}-(n-1)r^{n}/(1-r),$$

which as *n*→*∞* approaches *r*/(1−*r*)^2^<2 for all *r*. Thus *S*, the expected number of matching positions, is less than 2, and hence the expected number of letter comparisons required for each extension in algorithm ACSMF-Simple is less than 3. ■

#### 

**Theorem 3**. *Given a patternxof lengthmdrawn from alphabetΣ*, *σ*=|*Σ*|, *a texttof lengthn*>*mdrawn fromΣ*, *and an integer thresholdk*<*m*, *algorithm*ACSMF-simple*requires average-case time*$O((1+\frac{\text{km}}{\sigma^{\frac{2m-1}{2k+4}}})n)$*and space*O(m)*to solve Problem 2.*

*Proof.* By Fact 2, computing $E_{\ell }^{k}$ and $E_{r}^{k}$ for each occurrence of a fragment requires time O(kOcc). Therefore algorithm ACSMF-simple requires average-case time $O((1+\frac{\text{km}}{\sigma^{\frac{2m-1}{2k+4}}})n)$. The required space is reduced to O(m) since Step 4 of Algorithm ACSMF is not performed. ■

#### 

**Corollary 2**. *Given a patternxof lengthmdrawn from alphabetΣ*, *σ*=|*Σ*|, *a texttof lengthn*>*mdrawn fromΣ*, *and an integer threshold*$k=O(m/\underset{\sigma }{\log}m)$, *algorithm*ACSMF-simple*requires average-case time*O(n).

In practical cases, algorithm ACSMF-simple should be preferred over algorithm ACSMF as (i) it has less memory requirements (see Theorem 3); and (ii) it avoids the construction of a series of data structures (see Section 3 in this regard).

## Edit distance model

Algorithm ACSMF-simple could be easily extended for approximate circular string matching under the *edit distance* model (for a definition, see [10]). Since each single-letter edit operation can change at most one of the 2*k*+4 fragments of *x*^′^, any set of at most *k* edit operations leaves at least one of the fragments untouched. In other words, Lemma 2 holds under the edit distance model as well [27]. An area of length O(m) surrounding each potential occurrence found in the filtration phase (Steps 1-3 of algorithm ACSMF) is then searched using the standard dynamic-programming algorithm in time $O(m^{2})$[28] and space O(m)[29]. Since the expected number *Occ* of occurrences of the 2*k*+4 fragments is $O(\frac{\text{kn}}{\sigma^{\frac{2m-1}{2k+4}}})$, the average-case time complexity becomes $O((1+\frac{km^{2}}{\sigma^{\frac{2m-1}{2k+4}}})n)$ and the space complexity remains O(m). When $k=O(m/\underset{\sigma }{\log}m)$, the average-case time complexity is O(n).

## Experimental results

We implemented algorithms ACSMF and ACSMF-Simple as library functions to perform approximate circular string matching with *k*-mismatches. The functions were implemented in the C programming language and developed under GNU/Linux operating system. They take as input arguments the pattern *x* of length *m*, the text *t* of length *n*, and the integer threshold *k*<*m*; and then return the list of starting positions of the occurrences of the rotations of *x* in *t* with *k*-mismatches as output. The library implementation is distributed under the GNU General Public License (GPL), and it is available at http://www.inf.kcl.ac.uk/research/projects/asmf/, which is set up for maintaining the source code and the man-page documentation. The experiments were conducted on a Desktop PC using one core of Intel i7 2600 CPU at 3.4 GHz under GNU/Linux.

Approximate circular string matching is a rather undeveloped area. To the best of our knowledge, there does not exist an optimal (average- or worst-case) algorithm for approximate circular string matching with *k*-mismatches. Therefore, keeping in mind that we wish to evaluate the efficiency of our algorithms in practical terms, we compared their performance to the respective performance of the C implementation^a^ of the optimal average-case algorithm for multiple approximate string matching, presented in [17], for matching the *r*=*m* rotations of *x*. We denote this algorithm by FredNava.

Tables 1, 2, 3 illustrate elapsed-time and speed-up comparisons for various pattern sizes and moderate values of *k*, using a corpus of DNA data taken from the Pizza & Chili website [30]. As it is demonstrated by the experimental results, algorithm ACSMF-Simple is in all cases the fastest with a speed-up improvement of more than three orders of magnitude over FredNava. ACSMF is always the second fastest, while ACSMF-Simple still retains a speed-up improvement of more than one order of magnitude over ACSMF. Another important observation, also suggested by Corollaries 1 and 2, is that the ACSMF-based algorithms are essentially *independent* of *m* for moderate values of *k*.

**Table 1:** **Elapsed-time and speed-up comparisons of **FredNava**, **ACSMF**, and **ACSMF-Simple** for *n *** **=1MB**

		**Elapsed Time (s)**			**Speed-up of ACSMF-Simple**	
***m***	***k***	FredNava	ACSMF	ACSMF-Simple	FredNava	ACSMF
100	5	1.63	0.40	0.06	27	7
200	5	6.77	0.40	0.05	135	8
300	5	16.84	0.41	0.05	337	8
400	5	31.99	0.41	0.05	640	8
500	5	53.26	0.41	0.05	1065	8
600	5	81.35	0.41	0.05	1627	8
700	5	116.24	0.41	0.05	2325	8
800	5	158.73	0.41	0.06	2645	7
900	5	206.43	0.42	0.06	3440	7
1000	5	264.84	0.41	0.06	4414	7
100	10	1.65	0.43	0.05	33	9
200	10	6.94	0.40	0.05	139	8
300	10	16.55	0.41	0.05	331	8
400	10	31.70	0.40	0.05	634	8
500	10	53.11	0.41	0.05	1062	8
600	10	81.04	0.40	0.05	1620	8
700	10	116.25	0.41	0.06	1937	7
800	10	158.1	0.41	0.06	2635	7
900	10	207.33	0.41	0.05	4146	8
1000	10	264.11	0.41	0.05	5282	8
100	15	1.65	0.42	0.06	28	7
200	15	6.91	0.41	0.06	115	7
300	15	16.45	0.41	0.06	274	7
400	15	31.48	0.41	0.05	630	8
500	15	52.55	0.41	0.05	1051	8
600	15	80.46	0.41	0.05	1069	8
700	15	115.86	0.41	0.06	1931	7
800	15	157.81	0.41	0.06	2630	7
900	15	206.56	0.42	0.06	3443	7
1000	15	262.16	0.42	0.06	4369	7

**Table 2:** **Elapsed-time and speed-up comparisons of **ACSMF** and **ACSMF-Simple** for *n *** **=10MB**

		**Elapsed Time (s)**		**Speed-up of ACSMF-Simple**
***m***	***k***	ACSMF	ACSMF-Simple	ACSMF
10000	100	6.54	0.67	10
11000	100	6.69	0.70	10
12000	100	6.57	0.72	9
13000	100	6.64	0.74	9
14000	100	6.58	0.75	9
10000	300	6.54	0.69	9
11000	300	6.67	0.69	10
12000	300	6.64	0.68	10
13000	300	6.71	0.71	9
14000	300	6.63	0.72	9
10000	500	6.74	0.66	10
11000	500	6.58	0.67	10
12000	500	6.69	0.66	10
13000	500	6.66	0.67	10
14000	500	6.71	0.68	10

**Table 3:** **Elapsed-time and speed-up comparisons of **ACSMF** and **ACSMF-Simple** for *n *** **=50MB**

		**Elapsed Time (s)**		**Speed-up of ACSMF-Simple**
***m***	***k***	ACSMF	ACSMF-Simple	ACSMF
50000	500	45.71	4.33	11
51000	500	45.81	4.35	11
52000	500	45.73	4.37	10
53000	500	44.99	4.40	10
54000	500	45.05	4.40	10
50000	700	45.00	4.26	11
51000	700	44.79	4.18	11
52000	700	44.96	4.36	10
53000	700	44.83	4.32	10
54000	700	45.00	4.32	10
50000	900	46.79	4.32	11
51000	900	44.89	4.28	10
52000	900	45.06	4.33	10
53000	900	45.14	4.35	10
54000	900	44.81	4.12	11

## Conclusions

In this article, we presented new average-case algorithms for exact and approximate circular string matching. Algorithm ECSMF for exact circular string matching requires average-case time O(n); and Algorithms ACSMF and ACSMF-simple for approximate circular string matching with *k*-mismatches require time O(n) for moderate values of *k*, that is $k=O(m/\underset{\sigma }{\log}m)$. We showed how the same results can be easily obtained under the edit distance model. The presented algorithms were also implemented as library functions. Experimental results demonstrate that the functions provided in this library accelerate the computations by more than three orders of magnitude compared to a naïve approach.

For future work, we will explore the possibility of optimising our algorithms and the corresponding library implementation for the approximate case by using lossless filters for eliminating a possibly large fraction of the input that is guaranteed not to contain any approximate occurrence, such as [31] for the Hamming distance model or [32] for the edit distance model. In addition, we will try to improve our algorithms for the approximate case in order to achieve average-case optimality.

## Endnote

^a^ Personal communication with author.

## Competing interests

The authors declare that they have no competing interests.

## Authors’ contributions

CSI and SPP designed the study. CB, CSI, and SPP devised the algorithms. SPP developed the library and conducted the experiments. CB and SPP wrote the manuscript with the contribution of CSI. The final version of the manuscript is approved by all authors.
